# Comparison of Older and Younger Adults’ Attitudes Toward the Adoption and Use of Activity Trackers

**DOI:** 10.2196/18312

**Published:** 2020-10-22

**Authors:** Sunyoung Kim, Abhishek Choudhury

**Affiliations:** 1 School of Communication and Information Rutgers University New Brunswick, NJ United States

**Keywords:** older adults, technology acceptance, activity tracker, fitness tracker, mHealth, health care, quality of life

## Abstract

**Background:**

Activity tracking devices have significant potential in assisting older adults’ health care and quality of life, but this population lags behind in the adoption of these devices. While theoretical frameworks have been introduced to explain and increase the adoption of this technology by older adults, little effort has been made to validate the frameworks with people in other age groups.

**Objective:**

The goal of this study was to validate the theoretical framework of technology acceptance by older adults that we previously proposed through a direct comparison of the attitudes to and experiences of activity trackers in older and younger users.

**Methods:**

Semistructured interviews were conducted with 2 groups of 15 participants to investigate their experiences of using activity trackers. The recruitment criteria included age (between 18 years and 24 years for the younger participant group or 65 years and older for the older participant group) and prior experiences of using mobile devices or apps for activity tracking for 2 months and longer.

**Results:**

Our findings showed that the phase of *perceived ease of learning* as a significant influencer of the acceptance of activity trackers existed only in the older participant group, but this phase never emerged in the younger participant group. In addition, this study confirmed that other phases exist in both age groups, but 2 distinct patterns emerged according to age groups: (1) the *social influence* construct influenced the older participants positively but the younger participants negatively and (2) older participants’ exploration in the *system experiment* phase was purpose-driven by particular needs or benefits but for younger participants, it was a phase to explore a new technology.

**Conclusions:**

This study confirms the validity of the proposed theoretical framework to account for the unique aspect of older adults’ technology adoption. This framework can provide theoretical guidelines when designing technology for older adults as well as when generating new investigations and experiments for older adults and technology use.

## Introduction

### Background

Activity tracking devices that enable continuous monitoring of physical activities and physiological parameters have become widely available, allowing people to monitor their daily activity and overall health. People are now able to track their steps, heart rates, sleep patterns, and even engage in social forms of health tracking by using activity trackers [1]. With the collected data and presented information on these devices, not only can people gain insight into their daily activities but also be empowered to proactively manage and monitor health concerns, as physical activity helps reduce the risk of chronic diseases such as cardiovascular diseases, obesity, and diabetes [2].

The use of activity trackers by older adults is an area of particular research interest, since monitoring physical activity is a valuable parameter to define if persons are performing enough physical activities to prevent age-related chronic diseases or if they are manifesting early symptoms of those diseases [3]. However, there remains a notable digital divide between young adults and older adults. While over half of the Americans reported using a wearable fitness tracker at least once a day, only a little over 20% of the older adults owned an activity tracker in the United States as of 2016 [4]. In fact, low adoption of technology by older adults is not specific to activity trackers but is common with regard to any personal computing devices. While the adoption rates of computers and the internet by older adults are steadily increasing (from 12% in 2000 to 67% in 2016), these rates are significantly lower when compared with 90% of the general adult population using web-based services regularly [5]. Therefore, it is important to understand how older adults perceive and use new technology to meet their needs and to increase the adoption of new technology among older adults. To achieve this goal, studies have sought to understand how and why older adults maintain the use of new technology such as activity trackers and why they choose not to use or stop using this technology [6,7]. However, little comparative evidence exists with regard to the usage patterns and perspectives of older adults on new technology in a direct comparison with those of persons of other age groups.

Over the decades, technology acceptance models have been developed and refined to theoretically conceptualize the factors that influence the decision of whether to adopt new technology [8-13]. Within the context of technology adoption and the aging population, researchers have attempted to conceptualize older adults’ technology acceptance [14-16]. As part of this effort, we proposed a new framework to account for older adults’ acceptance of mobile technology for health care in our previous work [7], wherein *perceived ease of learning* had a significant influence on older adults’ technology acceptance behavior, which did not appear in the existing frameworks. This study aimed to validate this framework by directly comparing the attitudes to and experiences of activity trackers in older and younger users.

### Literature Review

#### Mobile Technology and Older Adults

Mobile technology is increasingly focused on the development of apps and tools to support health care, healthy living, and quality of life [17]. Wearable devices and other mobile technology for health care allow users to continuously track and manage health data without having to see their health care provider, such as diabetes management [18] and weight loss [19]. There is also a plethora of mobile apps for health care; as of 2019, there were over 45,000 apps for health care available for download from Apple app stores [20].

With regard to older adults and mobile technology, older adults are increasingly becoming savvy consumers of smartphone-based health solutions and information. With the increased desirability for aging in place, numerous technologies have emerged with the aim of supporting aging-related health concerns, including Alzheimer and dementia care [21], palliative care [22], monitoring fall risks [23,24], and osteoarthritis [25]. Moreover, research has shown that older adults hold positive views toward technology and have taken the steps for technology adoption [26,27]. For instance, Puri et al [28] showed that older adults were mostly accepting wearable activity trackers once they had a clear understanding of its value for their lives, and Preusse [29] showed that the adoption of activity trackers can be increased by addressing the barriers to acceptance. Despite the potential benefits and the increasing interests in mobile technology for health care, their adoption rates among older adults are still low [30]. Thus, researchers have extensively investigated how and why older adults decide to adopt and use mobile technology and why they choose not to use or stop using it. For instance, Lee and Coughlin [31] reviewed studies of older adults’ technology acceptance and identiﬁed factors that are critical for older adults’ acceptance of technology, including value, usability, affordability, accessibility, technical support, social support, emotions, independence, experience, and conﬁdence. However, relatively little effort has been put to directly compare older adults’ adoption of a new technology with those of the younger populations, with few exceptions [32,33].

#### Technology Acceptance Models for Older Adults

Technology acceptance models have been developed and refined over last couple of decades to explain technology adoption practices of different user groups [34] in various contexts [35] since the advent of foundational models, that is, Technology Acceptance Model [8] and the Unified Theory of Acceptance and Use of Technology [13].

Extending these models, researchers have sought to conceptualize older adults’ technology acceptance practice, though there are only few [14,36,37]. As part of this effort, we previously proposed a new theoretical framework to explain older adults’ acceptance of mobile technology for health care as an extension of the predecessor theories by investigating the experiences and perspectives of 2 groups of older adults who were aged 60 years or older: technology adopters and nonadopters [7]. This framework introduced the perceived effort of *learning* as a significant obstacle for older adults’ technology acceptance, which has been noted in prior research but has never been incorporated into any prior models of technology acceptance. For instance, Heart and Kalderon [38] suggest that special attention needs to be paid to teaching and training senior citizens to use new technology, and Yusif et al [39] pointed out lack of training as an area of concern in older adults’ technology adoption. In Klimova and Poulova’s literature review [15], they found that the existing technology acceptance models are suitable as the foundational theoretical basis for empirical studies, but more attention should be paid to forms of training for older adults. While these empirically grounded works are critical, there is no theoretical model that includes learning as an important phase of older adults’ technology acceptance.

**Figure 1:**
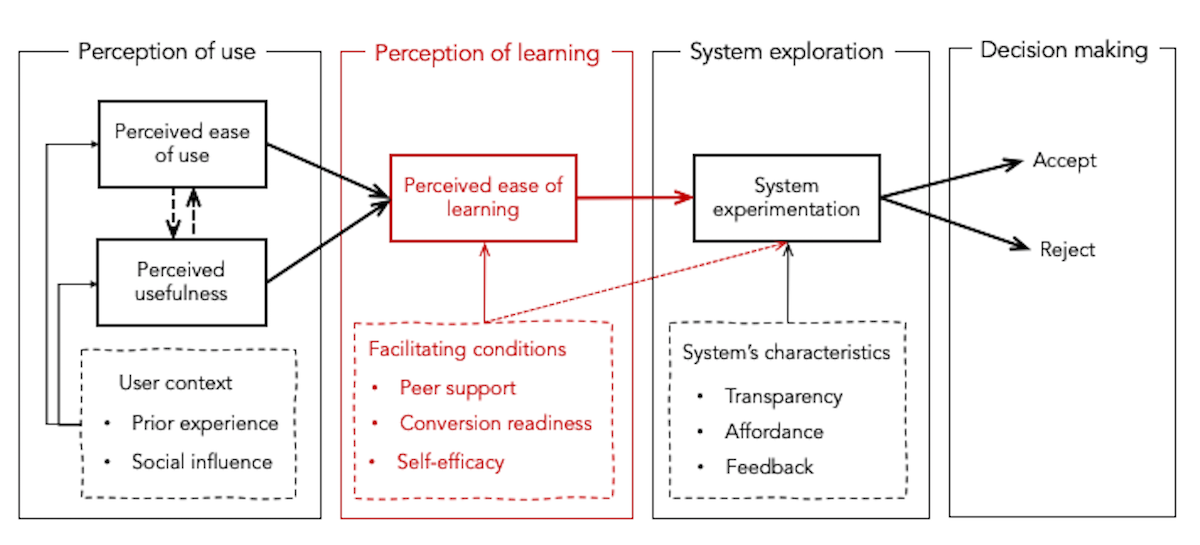
The proposed procedural model for older adults’ acceptance of mobile technology for health care. The red-boxed section is a new phase with accompanying constructs that is proposed to be crucial for older adults.

The theoretical framework we proposed comprises 4 phases, that is, (1) *perception of use*, the phase in which a user forms the intention to use a system; (2) *perception of learning*, the phase in which a user forms the intention to learn a system; (3) *system experimentation and exploration*, the phase in which a user explores and experiments with a system, and (4) *decision making*, the phase in which a user decides whether to accept or reject a system (Figure 1). This framework suggests that availability of facilitating conditions, including *peer support, conversion readiness,* and *self-efficacy*, is critical for older adults to take the first step into the digital world of *learning a new technology*, thereby echoing prior work [36,40]. While Davis [9] previously highlighted learning as an important construct to account for technology acceptance, he regarded “ease of learning” as a substratum of the ease of use construct, while our framework proposed that perceptions about use and learning are not necessarily related. Our previous finding demonstrated a clear distinction between “perceived ease of learning” and “perceived ease of use” among older participants; older adults tend to think that the device might be easy to use for young people but not necessarily for them. Thus, they tend to give up learning new technology regardless of it being perceived as useful [7]. The limitation of our proposed framework is that it has not been validated with the young population to assure its unique application in older adults. Further, there has been no research, to the best of our knowledge, that has evaluated the validity of any existing models with people in other age groups. Therefore, this study aims to validate our framework for older adults’ acceptance of mobile technology by conducting a comparison study with people in different age groups.

## Methods

### Data Collection

This study employed semistructured interviews and short questionnaires with 2 groups of 15 participants. The recruitment criteria included age (between 18 years and 24 years for the younger participant group or 65 years and older for the older participant group) and prior experiences of using mobile devices or apps for activity tracking for 2 months and longer. Semistructured interviews explored topics related to their everyday experiences of using activity trackers, including how they acquired the device, experiences of learning and using, resources for support, and when applicable, reasons for attrition. A questionnaire was administered prior to the interviews to record demographic information and the type of device the participant was using or has used before. These questionnaires were used as prompts to supplement the interview questions.

The first group, that is, “younger participant group” consisted of 15 college students and the second group, that is, “older participant group” consisted of 15 older adults. The younger participant group consisted of 10 males and 5 females with a mean (SD) age of 20.1 (1.5) years (age range, 18-24 years). Their mean (SD) duration of use of the device was 19.3 (17.6) months (range, 2 months to 6 years). They were recruited through flyers posted at the university’s student centers and the mailing lists. They were using a variety of activity tracking devices, including smartwatches, wristband-type activity trackers, and mobile apps for health care. The older participant group consisted of 4 males and 11 females aged 65 years or older with a mean (SD) age of 71.5 (6.3) years. Their mean (SD) duration of the use of the device was 38 (19.3) months (range, 2 months to 6 years). They were recruited through flyers posted at local libraries and senior centers. Device usage in this group, unlike that in the younger participant group, gravitated toward wristband-type activity trackers (eg, Fitbit) and mobile apps for health care (eg, the iPhone’s Health app) and nobody used smartwatches (see Table 1 for demographic information on the participants). Each interview lasted approximately an hour and participants were compensated for their participation. All interactions were audio recorded and transcribed. The study was reviewed and approved by the institutional review board.

**Table 1:** Demographics of the participants and the devices they used.

Younger (Y) participant group					Older (O) participant group				
ID	Gender^a^	Age (years)	Type of device	Duration of use	ID	Gender	Age (years)	Type of device	Duration of use
Y1	M	20	Fossil smartwatch	1 year	O1	F	89	Fitbit	3 years
Y2	F	19	Fitbit	2 years	O2	F	69	Fitbit	4 years
Y3	M	21	Frontier smartwatch	2 years	O3	F	69	iPhone Health app	4 years
Y4	M	19	Fitbit	2 years	O4	F	72	Fitbit	5 years
Y5	F	21	Fitbit	7 months	O5	F	69	Fitbit	6 months
Y6	M	22	Fitbit	1 year	O6	M	66	Fitbit	2 years
Y7	M	19	Apple watch	3 years	O7	M	78	Fitbit, iPhone Health app	3 years
Y8	M	24	Garmin smartwatch	2 months	O8	F	80	Fitbit	1 year
Y9	F	20	Fitbit	18 months	O9	F	71	iPhone Health app	3 years
Y10	F	21	Fitbit	2 years	O10	F	70	Fitbit, H-Band	5 years
Y11	M	18	Fitbit	3 months	O11	F	72	Fitbit	6 years
Y12	M	20	Fitbit, MyKronoz	1 year	O12	M	67	iPhone Health app	5 years
Y13	M	20	Fitbit, iPhone Health app	2 years	O13	M	69	iPhone Health app	2 months
Y14	M	19	Apple watch	18 months	O14	F	65	Fitbit	1 year
Y15	F	19	iPhone Health app, Apple watch, Lose it app	6 years	O15	F	67	Fitbit, Weight Watchers app, My Fitness Pal app	3 years

^a^Gender: male (M), female (F).

### Data Analysis

The interview data were analyzed using inductive and deductive approaches informed by grounded theory and other thematic analysis methods [41,42]. The themes and categories were identified deductively based on our proposed framework. Because the aspects and perspectives relating to *perceived learning* emerged as unique components that were critical to older adults’ technology acceptance but are not presented in existing models, these were the focus of the validation. Then, the interview transcripts were open-coded and analyzed both inductively to identify new themes that emerged from the data and deductively to validate the themes related to *learning*. Both authors read and discussed the interview transcripts and developed codes to describe important concepts that emerged directly from the data. We coded independently with frequent discussions to reach consensus. We then analyzed the data to verify the themes and to ensure we had reached data saturation until no new themes or concepts emerged.

## Results

### Definitions

We described our findings of the attitudes to and experiences of activity trackers in different participant groups by the first 3 phases of our framework, that is, *perception of use*, *perception of learning*, and *system experimentation.* The term *learning* used in this section refers to the acquisition of knowledge or skills by being taught from external resources and *tinkering* refers to a self-guided, hands-on, trial-and-error–based process to acquire knowledge or skills.

### Perception of Use

The first phase toward technology adoption in our framework is to formulate the perception about its use, which is influenced by its *perceived usefulness* and *perceived ease of use*. There were significant evidences to support the existence of these constructs from both participant groups.

#### Perceived Usefulness

Both participant groups acknowledged the perceived usefulness of activity trackers. All participants agreed on the potential utility of activity trackers to manage and improve health concerns (In the excerpts, “Participant O#” refers to the #th interviewee in the older participant group and “Participant Y#” refers to #th interviewee in the younger participant group).

…I think it’s a good thing because it helps you to understand how healthy you are and what you’re doing with yourself during the day to keep yourself healthy as you get older.

Participant O12

…In general, I think Fitbit is very useful...It really is a great device for tracking for people getting into shape and steps and anyone who is calorie counting.

Participant Y2

While the perceived “general” usefulness of activity trackers was unanimous across the groups, its perceived “personal” usefulness reflecting on one’s own potential benefits was divergent. Older participants described the perceived usefulness as a potential personal benefit to fulfill their own needs and deed, whereas younger participants perceived the devices to be useful for people other than themselves. This is not surprising since people generally become more vigilant about health concerns as they age, and young populations tend not to attend health care unless they have particular health problems. Prior work has shown that young adults have significantly lower rates of health care system utilization compared to older adults [43].

…I was excited because I could see that it was going to measure how many steps I was walking, how many times I was going up and down my steps, because I have about 14 steps in my house. So it was going to measure how many times I’m going up and down. I thought that was fascinating. You don’t realize how many times you go up and down a step and how many steps you take every day when you walk… But this makes me conscious of all of that.

Participant O8

…I don’t need all the fancy stuff about my health data, like how much I sleep, my nutrition, my laboratory results, and my reproductive health. Also, I definitely don’t need to have my health records on my phone… That’s not something I would want to do.

Participant Y6

While both *prior experience and social influence* emerged as significant constructs that influence the perceived usefulness in both groups, *social influence* was found to influence the perceived usefulness in different ways in different groups. For older participants, *social influence* positively impacted their perceived usefulness, as peers and other people in their close social network helped them discover and understand the potential utility of activity trackers. However, for some younger participants, *social influence* played a negative role, as an activity tracker was stereotyped as a tool for those with health concerns or weight management issues and thus using it was perceived to break a social norm of being healthy and active adolescents.

…My grandson gave it to me as a gift and I’ve had it about 2 or 3 years. He explained to me how it was working because he had one already and he thought that it would be a good idea for his old grandmother and his aunts to have one, so we all have one.

Participant O1

…You don’t want to be seen with a Fitbit in high school. It would make you look 30. A suburban soccer mom trying to get into shape, I assume, would love the Fitbit. Kids don’t want to do stuff like that. I didn’t really see anyone else with a Fitbit because people are going to be like oh, why is he tracking his steps? Is he like a soccer mom who just got it to get active?

Participant Y14

#### Perceived Ease of Use

The theme of *perceived ease of use* emerged as a significant factor to distinguish the adoption of activity trackers, though directions and perspectives were different in each group. All younger participants said that they would never expect any difficulty in interacting with new devices, while many older participants expressed a general fear of interacting with new technology, which is not an exception for the case of activity trackers.

…I know with computers you can lose everything. I mean if I lose something, I have no idea how to find it. Or, if I change a setting and I can’t find or go to where I want it to go anymore so that’s why it is intimidating for me.

Participant O9

…The general idea of it (Fitbit) and the main features that I would be working with all of them are very easy to understand. It’s very intuitive. And, it was pretty well organized. There wasn’t much I had to work on to use it.

Participant Y3

### Perception of Learning

Our findings confirmed that the *perceived ease of learning* phase exists only among older participants as a primary negative inﬂuencer in their adoption of activity trackers. It was evident that learning was perceived as a significant challenge for older participants. Older participants were hesitant to learn about using a new technology because they perceived that a new technology might be too difficult for them to learn, and some participants even thought that they are not capable of learning at all. Consequently, they refused to learn a new technology, regardless of its perceived usefulness. While a prior work by Renaud and Biljan [14] proposed *ease of learning* as an important construct in their model of seniors’ technology acceptance, our finding is different in that their notion of ease of learning occurs as part of the actual system use phase, whereas ours is *“perceived”* ease of learning that is formulated prior to the actual system experimentation and exploration. Meanwhile, there was no comment related to learning throughout the entire transcripts of the younger participants.

…At this age, to learn everything is not possible. I do emailing and certain things by myself, but I don’t want to learn everything because I may not be able to remember all that. But, certain things, if it is required for my Fitbit, I try to learn that. I will have to catch up with my grandson.

Participant O8

…I know technology is useful, but I don't make an effort to learn it. If you go to the phone company, they’ll help you… I'm afraid to touch buttons because I might throw the whole thing out of whack. I just feel like I can't do it.

Participant O11

Our framework has 3 constructs that were proposed to influence older adults’ *perception of learning* (and using) a new technology, that is, *peer support*, *conversion readiness*, and *self-efficacy*. The findings from this study confirmed that all 3 constructs have a significant influence on older participants’ perception of learning how to use activity trackers but none of these emerged in the data of the younger participant group. The first construct, *peer support,* refers to support from people in a close social network. Our findings confirmed that older participants rely primarily on *peer support* when interacting with activity trackers for the first time.

…My husband shows some functions (of Fitbit) to me. Then, I understand, I operate, and I work with that. After a few hours or few days, however, I forget that, and I will again ask him if he shows me again. It was not that easy to be familiar with that.

Participant O2

…I had to take help of my grandson to figure out how to set this up. Because this Fitbit is installed by my grandson and he knows when he goes to fix. I don’t know.

Participant O6

However, receiving support from other people was not something that older participants were always in favor of. In fact, they wanted to avoid seeking help from other people, if possible, which echoes the findings of prior work [7]. Researchers have found several reasons for older adults to be hesitant in receiving support from other people; older people are unwilling to reveal their lack of knowledge [44], a generational attitude of self-sufficiency exists, and the older adults prefer keeping their problems to themselves [45], or they do not want to bother people or interrupt people at what they consider to be crucial times [46]. While our findings did not demonstrate all these reasons, at least it was obvious that older participants tried to minimize seeking help from other people as much as possible, but most of the times, support from others was inevitable for them at least in the first few interactions with an activity tracker.

…Unfortunately, as much as I hate to admit, I will ask my friend for help. I really don't like asking him because I want to know how to do it on my own. But if I'm really stuck, then I'll ask him and then I can continue.

Participant O5

…I don’t like to bother my son too often because he’s very busy at work. So, when we see him maybe on Sunday morning at brunch where we get together for breakfast or something, I’ll ask him.

Participant O7

The second construct, *conversion readiness,* refers to the degree to which a person is ready to accept a new thing. Prior work demonstrated that older adults are resistant to changing their current practices regardless of how useful a new technology is because they are set to their own ways of doing things without the use of technology [7]. Our findings confirmed that this construct exists among older participants, negatively influencing the intention to learn and adopt a new technology. Since they were satisﬁed with the current way of doing things, they did not even attempt to ﬁnd out about the capabilities or benefits of new technologies, all of which did not appear among younger participants.

…I think the people and my friends at this age are more satisfied with what they have. I think, on average, the youngsters are enthusiastic with having more and more and more. That is the difference between those youngsters and we the people in the age ranges of 60 and 65 years. We are happy with what we have and what is needed, that’s it.

Participant O11

The third construct, *self-efficacy,* refers to the degree to which a person believes to be capable of accomplishing a task. Our findings confirmed prior work that older participants lack *self-efficacy* in a new technology, which negatively influences their intention to learn how to use a new technology [29]. When a technology did not operate properly, older participants blamed themselves for the problem, which resulted in feeling “scared” or “afraid” of using a new technology. Again, these tendencies never emerged in the data of the younger participant group.

…I’m afraid to set it up myself because then I might mess up something else. I’m afraid if I enter something and everything gets messed up. So, I would not try it on my own. That’s why it’s always good to watch my son set some of the stuff up for me.

Participant O15

While *perceived ease of learning* was found to be a significant challenge for older participants’ adoption of a new technology, they quickly became its active users once they successfully overcame this barrier. Several older participants who experienced difficulty in using activity trackers in their first acquisition reported that they now “feel comfortable” with using activity trackers because they “know what to do now.”

…At first, it was difficult to navigate through it because there is like, you press this, you get this menu and then you get this menu and then you get if you wanted to enter information, then you have to do all these things. But now I learned all and feel comfortable with using it.

Participant O4

…At first, I was paranoid, scared, whatever but after doing it and asking questions a couple of times, maybe 3 or 4 times, and it’s the same thing over and over. But now, I don’t have to keep bothering anybody what to do anymore. I know what to do now.

Participant O9

### System Experimentation

The *system experimentation* phase was confirmed to exist in both groups, though its pattern was different. Younger participants expressed a strong propensity to explore or tinker with a new technology rather than learning it when they first interacted with it as part of their efforts to figure out the features and functionalities of a device. A few younger participants searched information on the internet about how to use the device, but most of them jumped right into exploring and experimenting the features in their first interaction with it. Such explorations led to serendipitous discoveries of new functions and how to operate the features. Exploration and tinkering played as a key theme in which younger participants deepen their knowledge of the device. Numerous comments were received that demonstrated younger participants’ practices of tinkering with or exploring a device when they first acquired it throughout the entire transcript.

….I didn't read the manual. I just synced it (Fitbit) up with my phone and started using it. When I was looking for something, I could figure out myself or looked it up online.

Participant Y2

…I just messed around with it. I started play around with the features and see what other stuff it did by just pressing the buttons on the app, like the different icons, to see what I can do. Like I found out about that alarm menu in there.

Participant Y6

…I was toying around with all the features and such. I just press around the different icons to see what I can do. Like I found out about that alarm menu and there were a bunch of other options...I fiddled around with it for about 15 minutes, but I wouldn’t say there are any difficulties or complications with the device. So that’s what I did the first couple times I used the Fitbit.

Participant Y8

Older participants also commented on their practices of exploring a new technology as part of an attempt to find new technologies. However, their exploration patterns were distinct from those of the younger participants in that the older participants’ exploration was purpose-driven by particular needs or identified benefits, while younger participants’ exploration was more of serendipitous and random experimentation.

…There’s a lot of functions on it that I don’t even understand. At my age, I don’t explore that much but whatever I want to do, I try to investigate and find out like as I said the email, the—the phone, the texting, and the health part of it—that’s mainly what I use all the time. And that’s the benefit from having it, why I really wanted to have another one when the first one broke.

Participant O12

## Discussion

### Principal Findings

Our findings validated significant differences in the process through which people in different age groups accept or reject a new technology, using an activity tracking device as an exemplar. The phase of *perception of learning* existed among older participants as a significant influencer of their technology adoption but did not exist in younger participants. While older participants exhibited needs for some form of support for learning after acknowledging the *perception of use* but before *system exploration*, younger participants started to explore and tinker with activity trackers in their first acquisition of an activity tracker. This tendency can be explained by the fact that today’s generations of older adults have not grown up using the contemporary personal technologies since their childhood; therefore, they are not familiar with the technologies [7]. Thus, there might be a natural confounding factor associated with age and experience, since “today’s older adults are exposed to these technologies at a different point in their lives than today’s young adults” [47]. Our findings suggest that this natural confounder results in the emergence of *perception of learning* a new technology as a unique phase to facilitate older adults’ technology acceptance. The learning point of view is important because there will always be new technologies and new generations of older adults who have to learn how to use these. Therefore, in the development of new technologies, the learning perspective should be considered crucial to avoid exclusion of users of older groups.

Even though the phase of *perception of learning* was a significant challenge for older participants, it was not difficult to overcome. During the learning phase, older participants exhibited a strong reliance on *peer support* to either learn or get away from learning how to use an activity tracker, most of which successfully turned their final decision making into acceptance. This implies that it is crucial to provide older adults with easy access to facilitating conditions to lessen their tension and concern about learning a new technology [48]. While our data included only family members and friends as a resource of *peer support*, prior research has demonstrated a wide variety of resources within neighborhood groups and community groups that older adults can use to overcome learning-related difficulties, such as senior centers, local libraries, and local retail stores [49]. Offering classes lectured by older adult peers or peer-collaborative workshops through local community centers would be a way to lower older adults’ perceived effort of learning as well as helping them reduce the burden of asking for help to other people. Fostering older adults’ participation in such events will help technically isolated older adults ﬁnd potential peer support for technology adoption.

Lastly, this study showed that all phases of technology adoption except *learning* exist in both age groups, but the patterns of how some phases and constructs influence technology adoption were different in different age groups. First, our findings demonstrated that the *social influence* construct had a significant influence on technology adoption but varied in different age groups; social influence positively influenced older users’ technology, but it negatively influenced younger participants because an activity tracking device was negatively positioned for its use among some young generations. Second, the *system experiment* phase existed in both age groups, but the purpose was different; older user’s exploration was driven by particular needs or benefits after learning it, whereas younger users explored a new technology to tour available features and functionalities and to figure out how to use it as a first step into its use. This illustrates that more in-depth investigation and discussion of how a theoretical framework of technology adoption applies to different age groups, since the same factors can have a different (or even opposite) influence on technology adoption in different age groups.

### Limitations

The analyses presented in this paper are of a qualitative and explorative nature, providing in-depth insights into the issues older adults experience when using and learning to use activity trackers, in comparison to those experienced by college-age users. Small-scale qualitative studies have the advantage that they provide a rich picture of the ideas and experiences of the participants, but they are not able to provide a complete and representative picture of all the issues that are involved. Therefore, our results must be evaluated within the context of several study limitations. First, our sample size of patients was small (n=15 per group), and thus our participant pool may not be representative of a general population. In particular, all the younger participants were college students; therefore, they may not be representative of the entire young population. However, it is common in the sociology literature to regard college students as a representative of young adults when investigating age-related technology use practices since they are the major users of information and communication technologies [50,51]. In addition, other factors that might have influenced the results were not investigated, such as gender difference [52], difference by levels of technology expertise [53], or difference by the duration of device use [54]. In particular, further research would be helpful to explore the perspectives by usage durations since our participants had varying durations of activity tracker use; our data relied on participants’ memory, and memory could change over time. Lastly, all participants were recruited from an eastern metropolitan area of the United States. Therefore, our results may not generalize to the larger population of participants.

### Conclusion

In an aging society, technological advances can have a positive impact on promoting the quality of later life. An activity tracking device is a type of electronic wearable device that holds significant potential in assisting older adults’ health care by allowing to monitor and track health-related metrics. However, this population still shows slow rates of its adoption. While theoretical frameworks have been introduced to explain and promote the adoption of technology for older adults, little effort has been made to validate the frameworks with people in other age groups. Thus, we previously proposed a theoretical framework that sought to explicate technology acceptance for older adults [7], and this study aimed to validate this framework by directly comparing the attitudes to and experiences of activity trackers in older and younger users.

Our findings confirmed that the phase of *perceived ease of learning* as a significant influencer on the acceptance of activity trackers existed only among older users, but it never emerged among younger users. In addition, this study confirmed that other phases exist in both age groups, but 2 distinct patterns emerged by age groups: (1) the *social influence* construct influenced older participants positively but the younger participants negatively, and (2) older participants’ exploration in the *system experiment* phase was purpose-driven by particular needs or benefits, but for younger participants, it was a phase to explore a new technology’s features and functionalities. Based on these findings, we confirmed the validity of our proposed theoretical framework to account for the unique aspect of older adults’ technology adoption. This framework can provide theoretical guidelines when designing a technology for older adults as well as when generating new ideas for investigations and experiments about older adults and technology use. We are hopeful that our findings will be useful toward expanding the knowledge and practices for leveraging emerging personal technologies to support the aging society.
